# Effect of shikonin on the proliferation and apoptosis of human ovarian cancer cell SKOV3: A protocol of systematic review and meta-analysis

**DOI:** 10.1097/MD.0000000000020450

**Published:** 2020-05-29

**Authors:** Dan-feng Zhang, Dong-xu Zhao, Xue-zhu Liu, Jing Li, Yu-hong Hu, Peng-hui Dou

**Affiliations:** a Department of Obstetrics and Gynecology; b Department of Chemotherapy and Radiotherapy, First Affiliated Hospital of Jiamusi University; c Department of Physiology, Jiamusi University School of Basic Medical Sciences, Jiamusi, China.

**Keywords:** effect, human ovarian cancer cell SKOV3, shikonin

## Abstract

**Background:**

This study will investigate the effect of shikonin on the proliferation and apoptosis of human ovarian cancer cell SKOV3 (HOCC-SKOV3).

**Methods:**

We will retrieve potential studies from inception to the March 1, 2020 in Cochrane Library, MEDLINE, EMBASE, Scopus, Cumulative Index to Nursing and Allied Health Literature, WANGFANG, and China National Knowledge In-frastructure. There are not restrictions related to the language and publication status. This study will include case-controlled studies (CCSs) or randomized controlled studies (RCSs) that examine the effect of shikonin on the proliferation and apoptosis of HOCC-SKOV3. Two researchers will independently identify literatures, extract data, and appraise study quality. Any disagreements will be resolved by discussion with another researcher. RevMan 5.3 software will be placed to perform statistical analysis.

**Results:**

This study will summarize the present evidence to test the effect of shikonin on the proliferation and apoptosis of HOCC-SKOV3.

**Conclusion:**

It will provide evidence to investigate the effect of shikonin on the proliferation and apoptosis of HOCC-SKOV3, and will supply reference for further study.

Systematic review registration: INPLASY202040146.

## 1 Introduction

Ovarian cancer (OC) is one of the most common malignant cancers among female population.^[1–3]^ Although a variety of treatments are available for OC, the 5-year survival rate is still not satisfied.^[4–6]^ It is reported that expression of shikonin is highly characteristic, not expressed in normal adult tissues, but expressed in many tumors, and it is closely associated to the malignancy of the tumor and the prognosis of the tumor.^[7–10]^ Recent studies found that survivin abnormally overexpresses in human OC tumors.^[11–14]^ Furthermore, several studies specifically focus on the human ovarian cancer cell SKOV3 (HOCC-SKOV3).^[15–17]^

Previous studies reported that shikonin affects the proliferation and apoptosis of HOCC-SKOV3.^[18–20]^ However, there is no systematic review addressing the effect of shikonin on HOCC-SKOV3. Thus, this study aims to investigate the effect of shikonin on the proliferation and apoptosis of HOCC-SKOV3. It will provide helpful clue for further research.

## 2 Methods

### 2.1 Study registration

We have registered this systematic review on INPLASY202040146, and it has been reported in accordance with the Preferred Reporting Items for Systematic Reviews and Meta-Analysis (PRISRMA) Protocol statement guidelines.^[21–22]^

### 2.2 Eligibility criteria

#### 2.2.1 Types of trials

We will include all case-controlled studies (CCSs) or randomized controlled studies (RCSs) that examine the effects of shikonin on the proliferation and apoptosis of HOCC-SKOV3.

#### 2.2.2 Types of subjects

This study will choose HOCC-SKOV3 as its research targets.

#### 2.2.3 Types of interventions

The reference intervention is shikonin for the management of HOCC-SKOV3 in the experimental group.

There are many alternative treatment options for HOCC-SKOV3 in the control group. However, studies used shikonin as a control intervention will not be considered.

#### 2.2.4 Types of outcome measurements

Primary outcome is proliferation and apoptosis of HOCC-SKOV3. Its proliferation is examined by cell viability test, and its apoptosis is detected by flow cytometry.

Secondary outcomes are HOCC-SKOV3 proliferation and apoptosis related-proteins expression. The proteins comprise of cyclin D1, CDK2, P18, p-Rb, Bcl-2, Bax, cleaved caspase-3, p-PI3K, and p-AKT.

### 2.3 Literature sources and search strategy

We will undertake a comprehensive electronic databases search in Cochrane Library, MEDLINE, EMBASE, Scopus, Cumulative Index to Nursing and Allied Health Literature, WANGFANG, and China National Knowledge in-frastructure from the beginning to March 1, 2020 without limitations related to the language and publication status. There is an example for search strategy of Cochrane Library (Table 1). Similar search strategies for other electronic databases will be built.

**Table 1:**
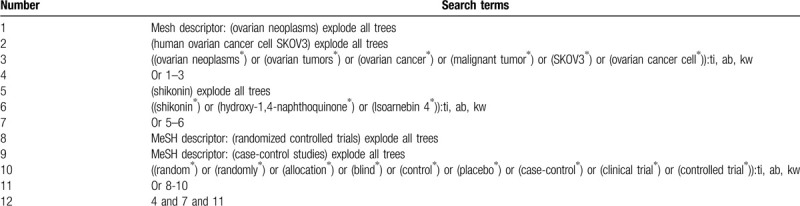
Search strategy for Cochrane Library.

In addition, we will search reports on relevant agencies, related conference abstracts, and reference lists of eligible studies.

### 2.4 Study selection

All research papers retrieved in the databases will be imported to EndNote X9 for data management. Two independent researchers will strictly scan titles and abstracts of searched articles according to the eligibility criteria. The full texts of potential articles will be obtained and will be carefully read against the inclusion criteria. If there is any difference between 2 researchers, we will seek the opinion of a third researcher. The study selection process will be presented in a PRISRMA flow diagram.

### 2.5 Data extraction and management

To extract data from eligible articles, a predefined data extraction sheet will be constructed to make sure all data collected integrity and relevance. Two researchers will independently extract data as follows: publication information (e.g., first author, year of publication, etc.), information of HOCC-SKOV3, study design, sample size, study methods, intervention, controls, and follow-up information. Any confusion will be cleared up by discussion with another researcher. If there is insufficient or missing data, we will contact the primary authors by e-mail or telephone.

### 2.6 Study quality assessment

Two researchers will separately appraise study quality of all eligible studies. We will settle any divergence in the evaluation through discussion with a third researcher. We will use Newcastle-Ottawa Scale to assess the study quality for CCSs, and will employ Cochrane risk of bias tool to evaluate study quality for RCSs.

### 2.7 Statistical analysis

This study will use RevMan 5.3 software, Cochrane Community; city, London; country, UK to implement statistical analyses. We will estimate treatment effects of dichotomous values as risk ratio and 95% confidence intervals (CIs), and continuous values as weighted mean difference or standardized mean difference and 95% CIs. *I*^2^ test is employed to examine heterogeneity across studies. *I*^2^ ≤ 50% indicates homogeneity, and a fixed-effects model will be applied, while *I*^2^ > 50% exerts significant heterogeneity, and a random-effects model will be placed. We will conduct a subgroup analysis to examine the possible sources of substantial heterogeneity.

### 2.8 Additional analysis

#### 2.8.1 Subgroup analysis

A subgroup analysis will be performed to examine the sources of significant heterogeneity according to the different types of studies, study quality, and intervention and comparators.

#### 2.8.2 Sensitivity analysis

A sensitivity analysis will be carried out to investigate the stability of study findings by eliminating studies with low methodological quality.

#### 2.8.3 Reporting bias

A funnel plot and Egger regression test will be identified to check reporting bias when >10 studies are included.

### 2.9 Dissemination and ethics

This study will not use individual patient data, thus, no ethical approval is required. We will publish this study on a peer-reviewed journal or conference presentation.

## 3 Discussion

Although several studies have examined the effects of shikonin on the proliferation and apoptosis of HOCC-SKOV3,^[18–20]^ there is still insufficient literature evidence on this topic. Thus, the primary objective of this study will explore the effects of shikonin on HOCC-SKOV3. This study will perform a qualitative and quantitative analysis of the overall merged data, and will hopefully to find helpful evidence for future studies. The restrictions of this study may be the heterogeneity of the methodological quality of primary studies, which may affect our findings.

## Author contributions

**Conceptualization:** Dan-feng Zhang, Xue-zhu Liu, Jing Li, Yu-hong Hu, Peng-hui Dou.

**Data curation:** Dan-feng Zhang, Peng-hui Dou.

**Formal analysis:** Dan-feng Zhang, Jing Li, Yu-hong Hu.

**Funding acquisition:** Peng-hui Dou.

**Investigation:** Peng-hui Dou.

**Methodology:** Dan-feng Zhang, Dong-xu Zhao, Xue-zhu Liu, Yu-hong Hu.

**Project administration:** Peng-hui Dou.

**Resources:** Dong-xu Zhao, Xue-zhu Liu, Jing Li, Yu-hong Hu.

**Software:** Dan-feng Zhang, Dong-xu Zhao, Jing Li, Yu-hong Hu.

**Supervision:** Peng-hui Dou.

**Validation:** Dan-feng Zhang, Dong-xu Zhao, Xue-zhu Liu, Jing Li, Peng-hui Dou.

**Visualization:** Dan-feng Zhang, Dong-xu Zhao, Jing Li, Peng-hui Dou.

**Writing – original draft:** Dan-feng Zhang, Xue-zhu Liu, Yu-hong Hu, Peng-hui Dou.

**Writing – review & editing:** Dan-feng Zhang, Dong-xu Zhao, Yu-hong Hu, Peng-hui Dou.
